# Comparisons of Rabbit Bone Marrow Mesenchymal Stem Cell Isolation and Culture Methods *In Vitro*

**DOI:** 10.1371/journal.pone.0088794

**Published:** 2014-02-18

**Authors:** Weidong Zhang, Fangbiao Zhang, Hongcan Shi, Rongbang Tan, Shi Han, Gang Ye, Shu Pan, Fei Sun, Xingchen Liu

**Affiliations:** 1 Department of Cardiothoracic Surgery, College of Clinical Medicine, Yangzhou University, Yangzhou, Jiangsu Province, China; 2 The Research Center for Translational Medicine, Yangzhou University, Yangzhou, Jiangsu Province, China; Rutgers - New Jersey Medical School, United States of America

## Abstract

Bone marrow mesenchymal stem cells (BMSCs) have great potential in tissue engineering and clinical therapy, and various methods for isolation and cultivation of BMSCs have been reported. However, the best techniques are still uncertain. Therefore, we sought the most suitable among the four most common methods for BMSC separation from rabbits. BMSCs were obtained from untreated whole bone marrow (BM) adherent cultures, 3 volumes of red blood cells (RBC) lysed with ammonium chloride, 6 volumes of RBC lysed with ammonium chloride, and Ficoll density gradient centrifugation. Then, isolated BMSCs were evaluated with respect to primary cell yield, number of CFU-F colonies, proliferative capacity, cell phenotype, and chondrogenic differentiation potential. Our data show that BMSCs were successfully isolated by all four methods, and each method was similar with regard to cell morphology, phenotype, and differentiation potential. However, BMSCs from untreated whole BM adherent cultures had greater primary cell yields, larger colonies, and the shortest primary culture time (P<0.05). Moreover, the 4^th^ generation of cultured cells had the strongest proliferative activity, the fastest growth rate and the most numerous cells compared with other cell passage generations (P<0.05). In conclusion, untreated whole BM adherent cultures are best for rabbit BMSC isolation and the 4^th^ generation of cells has the strongest proliferation capacity.

## Introduction

Stem cells have enormous utility in life science research. Specifically, bone marrow mesenchymal stem cells (BMSCs) which are adult stem cells derived from mesodermal cell lineages with self-renewable capacities and multi-directional differentiation potentials, are especially important [1]. Given appropriate culture conditions, BMSCs have been reported to differentiate into chondrocytes [2]–[3], osteocytes [4], adipocytes [5], endothelial cells [6], myocytes, cardiomyocytes [7], and even hepatocytes [8]–[9] and neurons [10], both of which are non-mesodermal in origin. BMSCs, as stem cells, have advantages of availability, culture expansion, low immunogenic properties [11]–[13], and ease of genetic manipulation, so they have wide use in numerous clinical applications, including tissue engineering [2]–[3], autoimmune disease [14] and myocardial infarction treatment [15], wound repair [16] and tissue regeneration [17].

Several methods are currently available for BMSC isolation and cultivation. Although separation methods that include immunomagnetic beads or flow cytometry generate BMSCs with higher purity, the expense, procedural complications, and the cell damage that occurs restricts their usage. At present, untreated whole BM adherent cultures, RBC lysis with ammonium chloride, and Ficoll density gradient centrifugation are the most common methods for obtaining BMSCs with acceptable purity, viability, and cost. However, the best method for isolating large numbers of BMSCs is uncertain. Peterbauer [18] reported that the highest BMSCs yields were obtained with RBC lysis, but this method was only compared to density gradient centrifugation. Horn and colleagues [19] compared RBC lysis with Ficoll density fractionation and untreated whole BM adherent cultures, reporting that BMSCs can be efficiently isolated by RBC lysis. Also, their technique was faster and could be standardized more easily for clinical applications. However, we found that untreated whole BM adherent cultures are more efficient than RBC lysis for isolating and purifying BMSCs. According to the literature [19], [24], 6 volumes of RBC lysis completely lysed erythrocytes and platelets in BM. However, 3 volumes of RBC lysis retained few erythrocytes, platelets, and some cell fragments. Through comparative analysis of different quantities of RBC lysis buffer, we further evaluated the effects of erythrocytes and platelets on proliferation of rabbit BMSCs *in vitro*. With this in mind, we compared untreated whole BM adherent cultures, 3 volumes of RBC lysis, 6 volumes of RBC lysis, and Ficoll density gradient centrifugation under the same conditions to find the best method for isolation and purification of rabbit BMSCs *in vitro*.

## Methods

### Bone marrow aspiration

**Figure 1:**
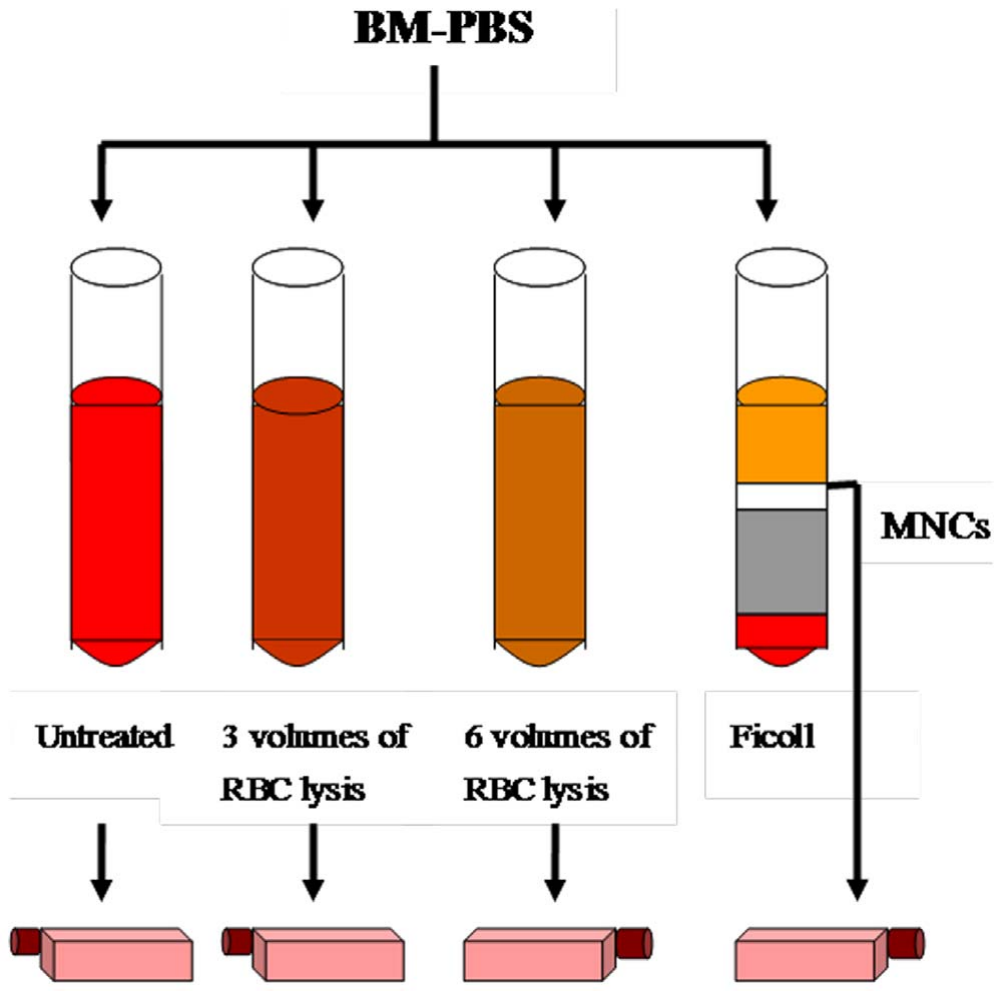
Scheme of BMSC isolation. BM-PBS aspirate was divided into four fractions for comparative isolation of BMSCs: 1) untreated whole BM aspirate, 2) 3 volumes of RBC lysis with ammonium chloride, 3) 6 volumes of RBC lysis, or 4) Ficoll density-gradient centrifugation. Finally, BM-PBS mixtures were added to the cell culture medium.

All animal procedures were approved by the Yangzhou University Laboratory Animal Care (Yangzhou, China) and this study was carried out in strict accordance with the Guidelines on the Care and Use of Laboratory Animals issued by the Chinese Council on Animal Research and the Guidelines of Animal Care. BM was harvested under aseptic conditions from the tibia and femur condyle of 6 anaesthetized New Zealand rabbits (0.75 kg, 1 month old, male). Then, a 32 ml mixture of BM aspirates and PBS partitioned in four equal fractions for simultaneous BMSCs isolation using 1) untreated whole BM aspirate, 2) 3 volumes of RBC lysis with ammonium chloride, 3) 6 volumes of RBC lysis with ammonium chloride, or 4) Ficoll density-gradient centrifugation (Figure 1).

#### 1) Isolation of BMSCs from untreated whole BM blood

The BM-PBS mixture (8 ml) was centrifuged for 5 min at 1,000 rpm (Labofuge 400R, ThermoFisher, Germany) and supernatant was removed. Pellets were washed with PBS and centrifuged again. To whole cell sediment, 8 ml culture medium was added consisting of 89% DMEM/F12 (HyClone, Utah, USA), 10% fetal calf serum (FCS, HyClone), and 1% penicillin/streptomycin/amphotericin B (Sangon Biotech, Shang hai, China).

#### 2) Isolation of BMSCs via 3 volumes of RBC lysis

The BM-PBS mixture (8 ml), was centrifuged for 5 min at 1,000 rpm and the supernatant was removed. The pellet was mixed with 3 volumes of RBS lysis buffer (Beyotime, Nantong, China) and incubated for 5 min at room temperature (RT) on a horizontal shaker. When the mixed liquid was light pink, five volumes of PBS was added to the mixture, and the mixture was centrifuged again. Subsequently cells were washed two more times with PBS and medium was added (qs to 8 ml) for cell culture.

#### 3) Isolation of BMSCs via 6 volumes of RBC lysis

BM-PBS mixture (8 ml) was centrifuged and the pellet was mixed with 6 volumes RBS lysis buffer and treated as previously described above.

#### 4) Isolation of BMSCs by Ficoll density-gradient centrifugation

BM-PBS mixture (8 ml) was allowed to stand for 15 min, and the supernatant was slowly transferred to a 15 ml centrifuge tube containing an equal amount of Ficoll-Paque® (Sigma, St Louis MO, USA). After 20 min of centrifugation (2,000 rpm) without braking, mononuclear cells (MNCs) were collected from the interphase (cloud-like cell layer), and filled to 8 ml with culture medium.

Four different cell suspensions as described above were also seeded in 96-well cell culture plates (Corning, New York, USA) and each cell suspension had 8-well replicates with 0.2 ml/well, and blank control wells were used. Four plates were replicated (I-IV). The cell suspensions were transferred to four 25 cm^2^ cell culture flasks (Corning) with 1.6 ml medium per flask. All cells were cultured at 37°C, 5% CO_2_ and 95% humidity. Non-adherent cells were carefully washed and the culture medium was changed after 48 h. Thereafter, the culture medium was replaced every 2-3 days, and the cells were subcultured at 90% confluence. The above experiment was repeated 6 times.

### Identification of BMSCs

#### Morphologic analysis

Cell shape and proliferation was analyzed daily with inverted phase contrast microscopy (CKX41SF, Olympus, Japan).

#### CD antigen analysis

Immunofluorescence analysis was performed as previously described [20]. Briefly, cells from passage 4 were fixed with 4% paraformaldehyde (CP, Shanghai, China) for 90 min, then, cells were washed with PBS for 5 min, incubated with 0.5% Triton X-100 (Sigma) at 37°C for 20 min, washed twice with PBS for 5 min each, blocked with 1% BSA at 37°C for 30 min, incubated with anti- rabbit antibodies: CD34-PE and CD44-PE (Bioss, Beijing, China), overnight at 4°C, and finally washed five times with PBS for 2 min each. Antibodies were replaced with PBS for negative control staining. CD34-PE and CD44-PE antibody distribution was observed with fluorescent microscopy (BX41TF, Olympus).

Flow cytometry was used to confirm surface antigen marker CD34 and CD44 expression. First, 1×10^6^ BMSCs were incubated with anti-CD34-PE, anti-CD44-PE for 30 min at 4°C in the dark. Labeled cells were washed, collected, and analyzed using the FACScan flow cytometry system (BD, Franklin Lakes, USA). Antibody was replaced with PBS for negative control staining.

### Chondrogenic differentiation assays

Chondrogenic differentiation experiments were performed with passage 4 cells from the four BMSCs isolation methods. First, 1×10^6^ cultured cells were plated in 6-well plates (Corning) and covered with chondrogenic medium, consisting of BMSC medium supplemented with 10 ng/ml transforming growth factor-β1 (TGF-β1, PeproTech, Princeton, USA), 100 ng/ml insulin-like growth factor-1 (IGF-1, PeproTech), 50 µg/ml ascorbic acid (Sigma) and 10 mmol/L dexamethasone(Sigma). The medium was replaced every 2-3 days. After 21 days, the four cell groups were analyzed by anti-rabbit Collagen II (Boster, Wuhan, China) staining overnight at 4°C, as described previously [18], [21].

### Analysis of four primary cells' proliferative activity by MTT assay

On day 4, 7, 10 and 13 after plating, an MTT assay was used to detect the proliferation activity as previously described [22]. In brief, cells were incubated with 20 µl 5 mg/ml MTT (Sigma) for 4 h. Then medium was removed and formazan salts were dissolved with 150 µl of dimethyl sulfoxide (DMSO, Sigma), and the absorbance was read at 570 nm with an automated microplate reader (BioTek, Highland Park, USA). All procedures were repeated 6 times and cell proliferation for all four cell culture types was analyzed.

### Proliferation assays for passaged cells

MTT (Sigma) assay was used to measure cell proliferation from different cell passages. Confluent 2^nd^, 3^rd^, 4^th^, and 5^th^ passage cells were trypsinized and re-suspended separately. All cell passages were counted and seeded at a density of 3,000 cells/well in 10 96-wells plates, with 8 wells for each passage. Blank controls were used. All cells were cultured at 37°C in a humidified atmosphere containing 5% CO_2_. The MTT assay was conducted at day 1, 2, 3, 4, 5, 6, 7, 8, 9 and 10, prior to treatment to establish a growth curve for differently passaged cells. Each experiment was repeated 3 times (N = 8/group).

### Statistical analysis

Data from the four primary cell cultures and the number of different cell passages are reported as means and standard deviations (SD). *p*<0.05 was considered statistically significant.

## Results

### Identification of BMSCs

#### Morphologic analysis

**Figure 2:**
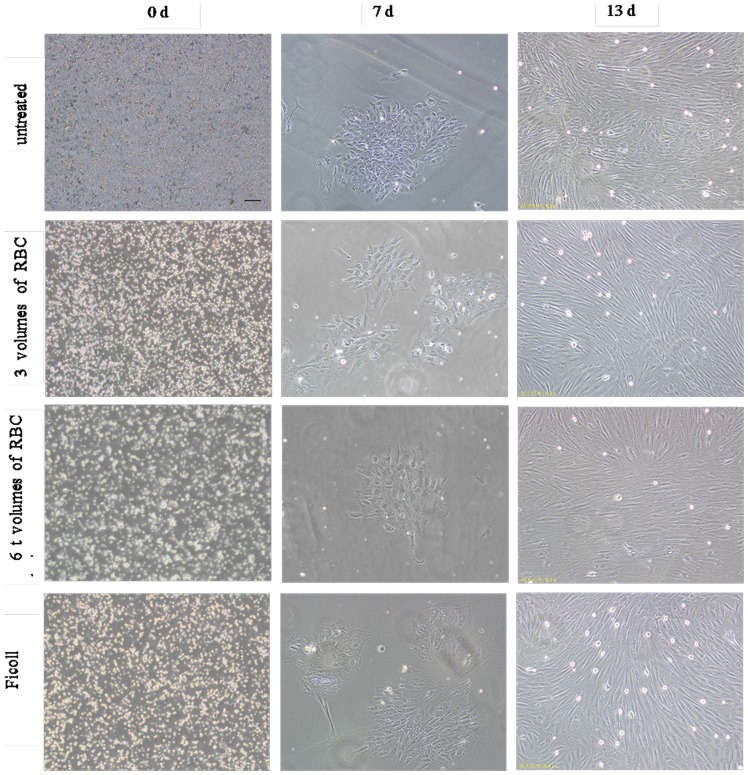
Morphologic analysis of BMSCs. Many RBCs were observed under phase-microscopy, after cells were seeded in cell culture flasks. RBCs in: untreated samples > after 3 volumes of RBC lysis > after 6 volumes of RBC lysis≈Ficoll samples. Fusiform adherent cells of four groups of BMSCs were observed after 24 h cultivation. Cells from four groups yielded many CFU-F colonies, when cells were cultured for 4–10 days, but the CFU-F count of the untreated sample was the greatest. At day 13, the four groups of BMSCs all had uniform spindle morphology, and reached about 100% confluence. Scale bar  = 100 µm, applies to all images.

RBCs were observed in all four groups after cells were seeded in cell culture flasks. The whole BM blood group had the most RBCs, followed by the group treated with 3 volumes of RBC lysis, the group treated with 6 volumes of RBC lysis, and the density gradient centrifugation group. Fusiform adherent cells were obvious when the four groups of BMSCs were cultured for 24 h. After two replacements of culture medium, many impure cells including RBCs, platelets and leucocytes in the culture were cleared. Cells also formed many CFU-F colonies after the 24-h incubation in all four groups. However, more CFU-F colonies were observed from untreated whole BM blood cultures compared with other culture methods. CFU-F colonies from untreated whole BM blood cultures formed earlier than cells isolated from other methods. Cell morphology was varied: spindle-shaped cells, and triangular, irregular, and unorganized cell arrangements were observed. At day 10, cells reached 80% confluence. At day 13, the cells had a uniform spindle shape, and reached ∼100% confluence (Figure 2). When cells were incubated for 15 days, they became senescent, with an irregular shape, cell atrophy, and more cell gaps. Six hours after confluent adherent cells were detached with 0.25% trypsin (Gibco, Carlsbad, USA) and re-seeded, some cells began to grow adherently. After 24 h, almost all cells adhered to cell culture flask, and after 10 days, cells reached 100% confluence.

#### Analysis of immunophenotype

**Figure 3:**
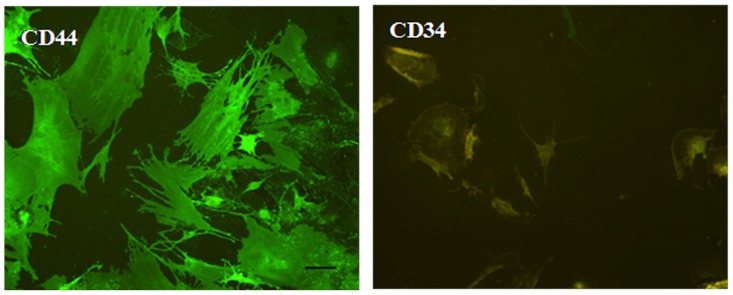
Immunofluorescence staining of BMSCs. CD44-PE was fluorescent green on the cell surface. CD34-PE was fluorescent brown, and this corresponded to BMSCs stained with antibody against CD44 and CD34, Scale bar  = 100 µm.

**Figure 4:**
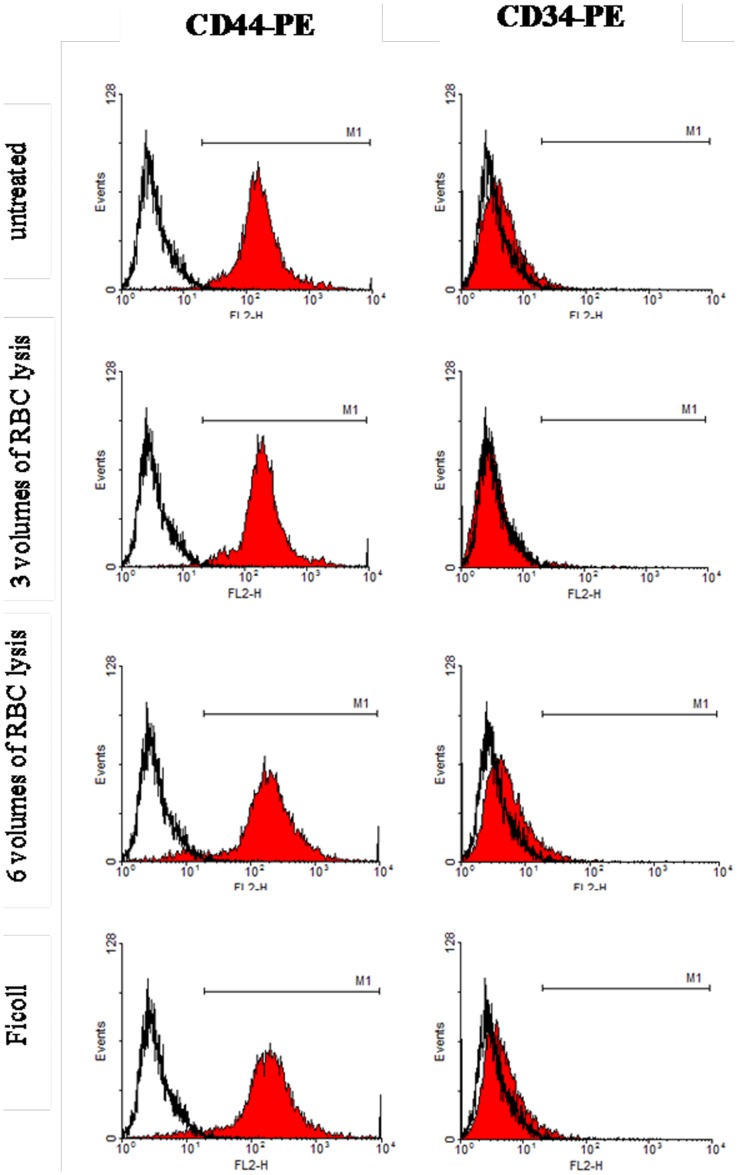
Flow cytometry. CD44 positive BMSCs were 98.12, 98.85, 93.32, and 94.32%, and CD34 positive cells were 3.25, 2.37, 5.31, and 3.50%, as shown by flow cytometry (autofluorescence is marked as a white filled histograms). There were no obvious differences among the four groups (*p*<0.05). Cells that expressed CD44 markers, but not CD34, correspond to BMSCs.

Immunofluorescent staining indicated that the CD44-PE cell marker fluoresced green on the cell surface under a fluorescent microscope. CD34-PE was fluorescent brown (not green). So, cultured cells were positive for CD44, but negative for CD34 (Figure 3). Flow cytometry was used to quantify BMSCs percentages, and CD44-positive cells were 98.12, 98.85, 93.32, and 94.32%, and the CD34-positive cells were 3.25, 2.37, 5.31, and 3.50%. These data confirmed that cells obtained from all four methods were BMSCs. There were no obvious differences among the four groups of BMSCs (Figure 4).

### Analysis of differentiation potential

**Figure 5:**
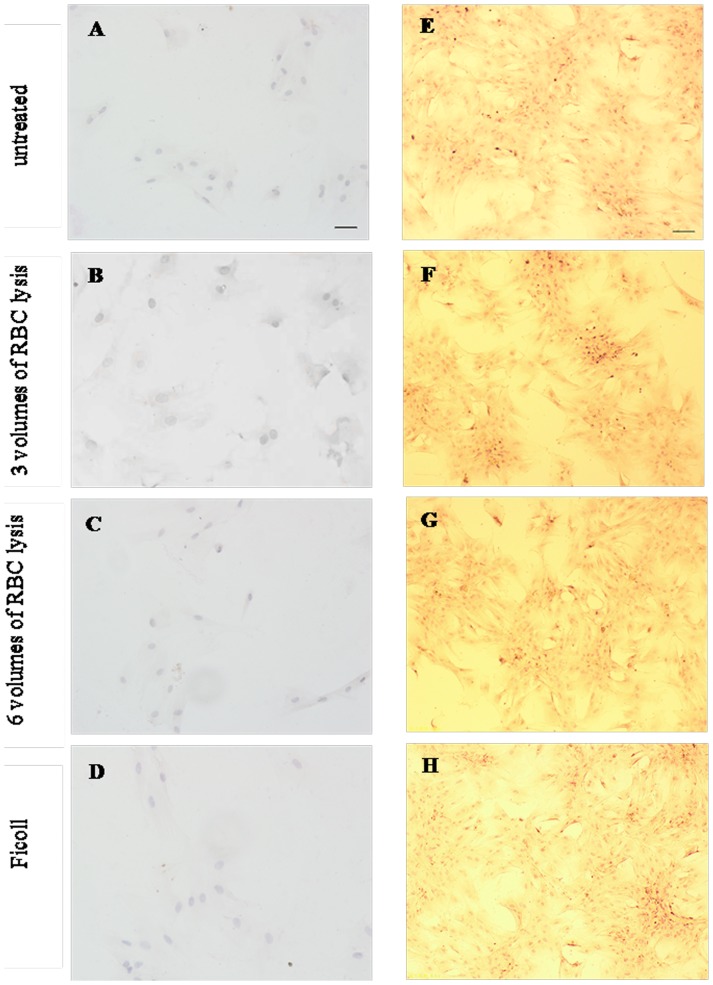
Chondrogenic differentiation of rabbit BMSCs isolated by four methods. Collagen type II immunostaining showed that the majority of BMSCs were differentiated into a chondrogenic lineage for 21 days (E–H), and were simultaneously cultured in control medium (A–D). Scale bar  = 50 µm, applies to A–D, and scale bar  = 100 µm, applies to E–H.

Collagen type II immunostaining confirmed that BMSCs isolated by four unique methods were equally suited for synthesis of high levels of type II collagen in chondrogenic differentiation cultures. As shown in Figure 5, no differences in differentiating potential were observed among the four uniquely isolated groups of BMSCs.

### Proliferative activity of primary cells

**Figure 6:**
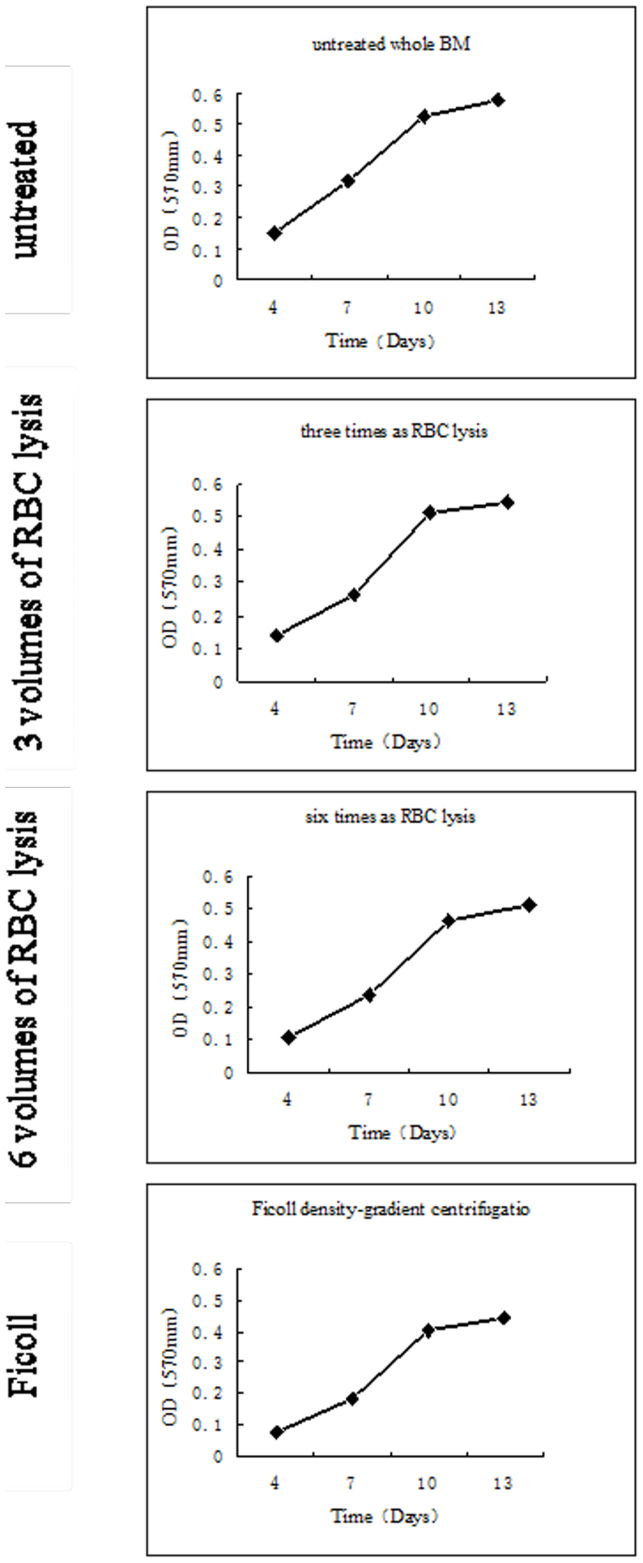
Comparison of four primary BMSC growth curves. All growth curves form four ell groups had an “S” form and no isolation method was shown to change cell growth.

**Figure 7:**
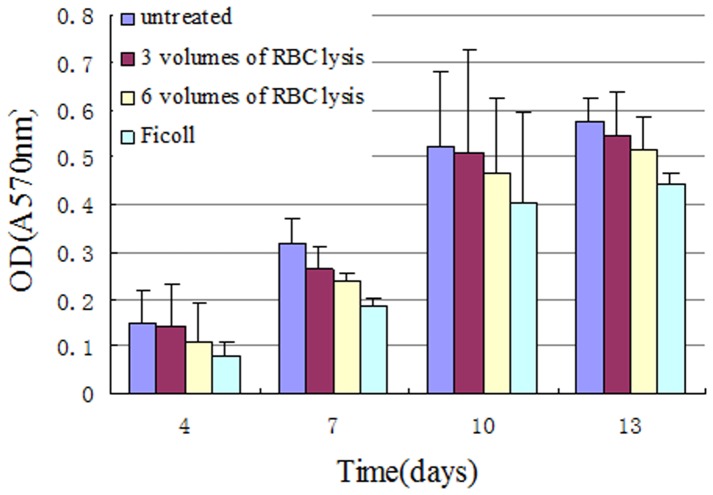
Cell proliferative activity among four primary cells. Cells from untreated whole BM blood proliferated better than the other 3 groups as shown by MTT assay. The MTT assay was conducted at the fourth, seventh, tenth and thirteenth day (*p*<0.05).

Analysis of average absorbances of cells isolated from four methods. All four groups of primary cells began to grow adherently at about 24 h, and the log growth phase of cells was stable around the 4^th^-9^th^ day. Growth curves of all four cell groups were “S”-type curves. Primary cells reached 80% confluence at day 10 and at day 13 cells were ∼100% confluent (lag phase). Statistically significant differences among the four primary BMSC growth curves were observed (Figure 6). Data show that untreated whole BM blood isolates were superior to the other 3 groups at the 4^th^, 7^th^, 10^th^, and 13^th^ day (*p*<0.05) (Figure 7).

### Passage cell proliferation assays

**Figure 8:**
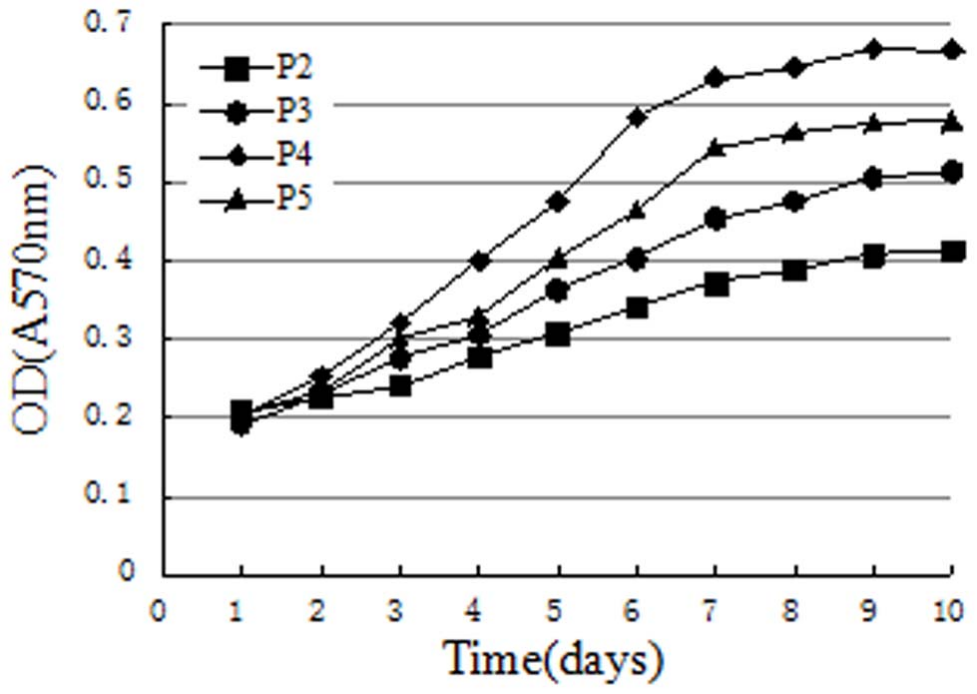
Cell proliferation assays for different cell passages. Cells from the fourth passage had the greatest proliferation, and second passage cells had the least proliferative ability (*p*<0.05).

The proliferation of the 2^nd^, 3^rd^, 4^th^, and 5^th^ cell passages were analyzed with an automated microplate reader (570 nm absorbance) at day 1-10. As shown in Figure 8, growth curves of passaged cells had an “S” shape and growing latent phases of passaged cells became stable at the 1^st^ to 2^nd^ day. The log phase of growth appeared about the 3^rd^ to the 8^th^ day and then cells reached the plateau The fourth and fifth passages of cells were ∼100% confluent at 7 days, and entered a lag phase. However, the second and third passages of cells entered the lag phase 2 days later. Thus, cells from the 4^th^ passage had the greatest proliferation ability (*p*<0.05), with a greater growth speed and cell quantity than other passages.

## Discussion

The process of purification and separation of BMSCs is crucial for further clinical trials, because untreated whole BM blood included erythrocytes, leukocytes, and platelets, but, the proportion of mononuclear cells was minimal. Isolation and purification of BMSCs from untreated whole BM blood is a common technique, growing cells in cell culture flasks until harvesting of adherent cells [23]. The RBC lysis method depends on the passage of NH_3_ and CO_2_ through the membrane, which are spontaneously converted to NH_4_^+^ in the cell and HCO_3_^-^ by carbonic anhydrase in the erythrocyte and platelet, respectively and thus could be the force behind a continuous flow of NH_3_ and CO_2_ entering the cell. This will change the osmotic fragility of erythrocytes and disrupt the membrane structure [24]–[25]. Lysis frequencies affect BMSC viability. Here, we compared 3 and 6 volumes of RBC lysis. Density-gradient centrifugation methods are based on the suspension density of BM, and the harvesting of mononuclear cell layers after centrifugation [26]–[28].

Dynamic cell morphology observations revealed that BMSCs grew in clusters with various morphologies. BMSCs had a uniform morphology at day 10–13, and were senescent at day 15. Therefore, subculturing is best when primary cells are cultured within 2 weeks.

Cell surface markers are key to identifying BMSCs [29] and our BMSCs preparations were positive for CD29, CD44, CD90, CD105, and negative for hematopoietic markers and endothelial markers such as CD14, CD34, CD45 and CD184. Immunofluorescence and flow cytometry confirmed that all cells from the four methods were positive for expressions of CD44 and negative for CD34, thus this confirmed BMSCs [30]–[32]. Data show that the four methods employed in the current investigation were not different with respect to cell purity.

Collagen type II is a marker specifically produced in cartilage cells, the expression of which increases during chondrogenic differentiation [33]. Here, we showed that the chondrogenic differentiation potential of expanded cells, as assessed by microscopic evaluation of the appearance collagen type II, did not differ among the four isolation methods.

We compared four isolation strategies of BMSCs from untreated whole BM, 3 and 6 volumes of RBC lysis, and Ficoll density-gradient centrifugation. We observed that untreated whole BM cultures were the most suitable and reliable methods of isolating rabbit BMSCs. BMSCs from untreated whole BM grew stably *in vitro*, proliferated rapidly, harvested large yields, and were not different with respect to cellular proliferation, morphology, immunophenotype, and differentiation potential. Advantages of untreated whole BM methods are convenience, low probability of microbial contamination, and a high ratio of cultivated cells. Therefore, this method is effective for large-scale experimental research. Our data also suggest that abundant erythrocytes and platelets in BM-PBS mixtures could affect BMSCs adherent growth, but that small amounts of erythrocytes and platelets promoted adherence. Evidence suggests that erythrocytes and platelets promote initial growth of BMSCs colonies, which might be attributed to various growth factors and an intact original BMSCs microenvironment [19], [34]–[35]. With RBC lysis and Ficoll density-gradient centrifugation methods, erythrocytes and platelets are completely removed, as well as growth factors that may affect BMSC proliferation. Also, frequent manual manipulation of cells may cause contamination and mechanical damage [27]. Analysis of two groups treated with RBC lysis confirm that 3 volumes of RBC lysis yielded greater proliferative BMSCs compared to 6 volumes of RBC lysis.

Analysis of 2^nd^, 3^rd^, 4^th^, and 5^th^ cell passages second, third, fourth, fifth passage cells proliferation, revealed that the growth curve of all cells passages cells had an “S” shape and that the logarithmic growth phase occurred at day 3 to 7. At 9 days, most cells were spindle-shaped and regular, and were approximately 100% confluent. Fourth generation BMSCs has a greater proliferation capacity than the other 3 generations, and the second generation was the weakest with respect to proliferation capacity.

In conclusion, untreated whole BM blood adherent culture methods offer a reliable, simple, and efficient method for isolation and purification of BMSCs. With this method the average colony size is larger compared with RBC lysis and Ficoll density-gradient centrifugation procedures. The BM blood adherent culture technique did not affect BMSC heterogeneity, differentiation potential, or biological activity compared to the other methods. So, this technique is most appropriate for experimental research and clinical applications of BMSC.
